# Case Report: Neonatal right atrial mass of uncertain etiology

**DOI:** 10.3389/fped.2025.1621500

**Published:** 2025-07-02

**Authors:** Libor Svoboda, Sabine Mank, Sabine Meier, Marcel Vollroth, Alexandra Kiess, Christian Schürer

**Affiliations:** ^1^ Department of Pediatric and Adolescent Medicine, University Hospital Jena, Jena, Germany; ^2^ Section of Pediatric Radiology, Department of Radiology, University Hospital Jena, Jena, Germany; ^3^ Department for Pediatric and Congenital Cardiac Surgery, Leipzig Heart Center, Leipzig, Germany

**Keywords:** neonatal cardiac mass, intracardiac thrombus, cardiac tumor, echocardiography in neonates, cardiac magnetic resonance imaging (MRI), pediatric cardiac surgery, histopathology in neonatal cardiology, case report

## Abstract

**Background:**

Neonatal intracardiac masses are rare and pose significant diagnostic and therapeutic challenges, particularly in differentiating thrombi from tumors.

**Case summary:**

We present the case of a preterm neonate with a right atrial mass of uncertain etiology. Multimodal imaging, including echocardiography and cardiac MRI, suggested the presence of a thrombus—consistent with thrombi being the most common type of intracavitary cardiac mass. As a result, primary anticoagulation therapy was initiated. However, after 16 days without significant change in the mass and given the high risk of embolization and the possibility of a benign tumor, surgical excision was performed. Histopathological analysis of the excised tissue could not definitively distinguish between an organized thrombus and a regressed benign neoplasm, although no malignant cells were identified.

**Conclusions:**

This case highlights the diagnostic uncertainty surrounding neonatal intracardiac masses and the limitations of imaging and pathology in achieving definitive diagnosis. A multidisciplinary approach and long-term follow-up are essential, particularly when the true nature of the mass remains unclear.

## Introduction

Neonatal intracardiac masses, including tumors and thrombi, are rare but represent significant diagnostic and therapeutic challenges. Primary cardiac tumors are identified in approximately 1 out of every 10,000 routine autopsies (0.01%) across all age groups (1). Primary cardiac tumors in neonates are extremely uncommon, with an estimated prevalence of 0.3 per 1,000 live births (2). The most common cardiac tumors in newborns are rhabdomyomas, associated with tuberous sclerosis in >50% of cases (1–3). The majority of cardiac tumors in children are benign (e.g., rhabdomyomas, fibromas, myxomas, hemangiomas, lipomas, or teratomas) (4–6). Primary malignant cardiac tumors in children are extremely rare (5, 6). The clinical presentation can range from asymptomatic to severe hemodynamic compromise, congestive heart failure, obstruction of the inflow or outflow tract of the heart, arrhythmias, tumor embolization or sudden death (5–8).

Intracardiac thrombi in neonates are rare but carry significant risks of morbidity and mortality (9). Neonatal thrombosis almost always occurs in the setting of identifiable risk factors (in over 95% of cases) (10). The most significant risk factors for thrombosis include central venous catheters, renal or cardiac diseases (e.g., low cardiac output, ventricle dilation, arrhythmias), polycythemia, asphyxia, septic or inflammatory conditions, and prothrombic disorders (10–12). Studies indicate that up to 90% of neonatal thromboses are catheter-related and ∼9% of infants with central lines develop thrombosis. Malpositioned catheter tips (e.g., too high in the atrium) further increase thrombosis risk (9).

Differentiating a cardiac tumor from an intracardiac thrombus is critical for guiding management (13). Clinically, tumors and thrombi can present as an unexpected mass on imaging, but their treatment diverges (surgical resection or observation for tumors vs. anticoagulation for thrombi). Because biopsy is rarely feasible in a neonate, noninvasive imaging is the cornerstone of diagnosis (14). Multiple imaging modalities – including echocardiography (transthoracic and transesophageal), cardiac magnetic resonance imaging (MRI), computed tomography (CT), and even positron emission tomography (PET) – each offer unique strengths in characterizing intracardiac masses. Transthoracic echo is the first step whenever an intracardiac mass is suspected or detected (15). It usually confirms the presence of a mass, its basic characteristics, and any urgent hemodynamic issues. If additional characterization is needed, cardiac MRI is generally the preferred second-line modality for neonates and children (16).

We report a case of a preterm neonate with a right-atrial mass that posed a diagnostic challenge. The mass was initially suspected to be a thrombus. When anticoagulant treatment proved to be ineffective and concerns of tumor possibility were raised, surgical excision was performed. Histopathological analysis of the excised tissue could not definitively differentiate between an organized thrombus and a regressed benign neoplasm. This case highlights the importance of multimodal imaging (echocardiography and MRI) and histological evaluation in the assessment of neonatal cardiac masses, as well as the challenges in reaching a definitive diagnosis—even with non-invasive multimodal imaging and tissue analysis. It also underscores the need for a multidisciplinary strategy and careful follow-up in such high-risk cases, especially when the mass's true nature remains uncertain.

## Case presentation

The patient was born at a gestational age of 28 weeks and 3 days, with a birth weight of 1,080 g (corresponding to the 35th percentile). The delivery was performed via cesarean section due to maternal preeclampsia, following a completed course of antenatal betamethasone therapy. Apgar Scores were 6/8/9. A prenatal ultrasound conducted during pregnancy did not reveal any cardiac anomalies. The patient was admitted to the neonatal intensive care unit for management of prematurity. Due to the respiratory distress syndrome, a single dose of surfactant was administered via less invasive surfactant administration (LISA) on the first day of life. The infant remained stable on continuous positive airway pressure (CPAP)/High-Flow therapy. The infant had not undergone umbilical vein catheterization. Instead, a peripheral epicutaneo-caval catheter was used for drug administration during the first week of life. The catheter was inserted via the saphenous vein into the inferior vena cava but did not reach the right atrium, as confirmed primarily by x-ray. On day three of life, a hemodynamically significant patent ductus arteriosus (PDA) was diagnosed and treated with ibuprofen. Follow-up echocardiography on day six confirmed successful PDA closure and showed no evidence of intracardiac masses or catheter malposition within the right atrium. The infant later developed mild bronchopulmonary dysplasia but had no other significant complications. However, during a routine echocardiographic examination prior to discharge at approximately 2.5 months of age, an incidental right atrial mass was detected, despite the absence of any cardiac murmur. A comprehensive diagnostic evaluation was undertaken:

### Echocardiography

**Figure 1:**
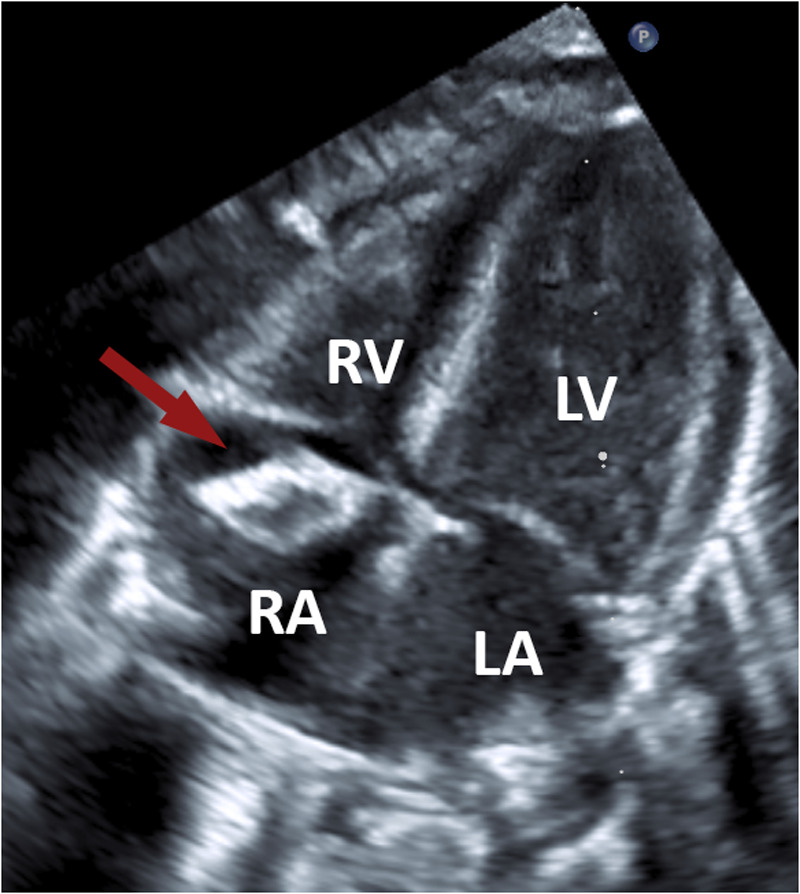
Transthoracic echocardiographic image demonstrating a mass (arrow) within the right atrium. RA, right atrium; LA, left atrium; RV, right ventricle; LV, left ventricle.

Transthoracic echocardiography revealed a highly mobile, inhomogeneous floating mass in the right atrium (Figure 1) attached to the interatrial septum, which did not obstruct valves or flow. The mass had echogenic rims and a relatively lucent center, with acoustic shadowing posteriorly. It prolapsed towards the tricuspid valve, without causing significant regurgitation or obstruction. From the echocardiographic findings, differential diagnosis was an organized thrombus vs. a cardiac tumor (e.g., myxoma, fibroma, hemangioma, teratoma). There were no signs of pulmonary hypertension, significant valve dysfunction or pericardial effusion. The biventricular function remained uncompromised.

### Cardiac MRI

**Figure 2:**
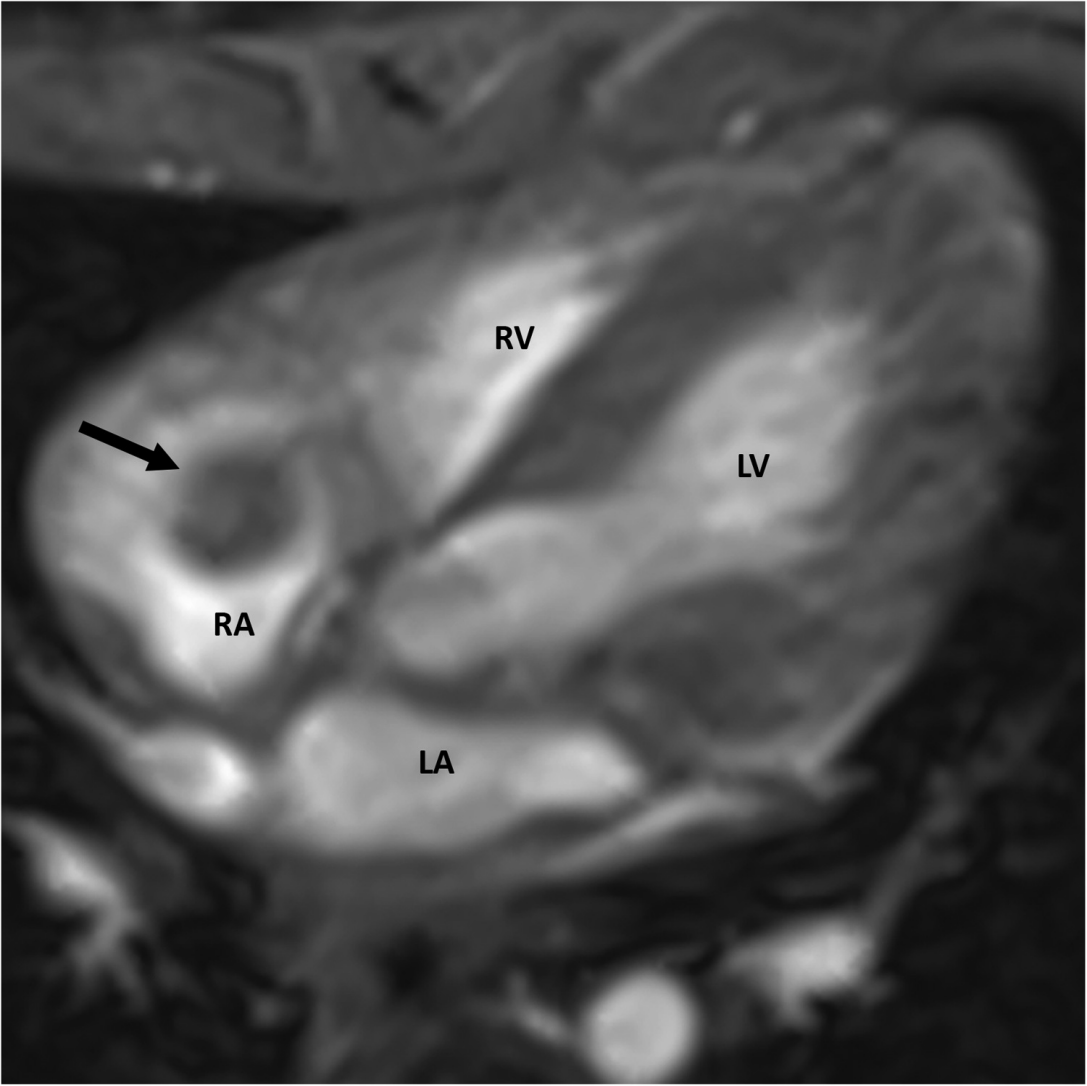
Cardiac MRI showing a well-defined mass (arrow) within the right atrium. RA, right atrium; LA, left atrium; RV, right ventricle; LV, left ventricle.

A cardiac MRI was performed for further tissue characterization. It demonstrated a round, well-defined hypointense structure (approx. 6 × 7 × 11 mm) in the right atrium (Figure 2), pedunculated and attached to the atrial roof. There was no evidence of intramyocardial extension of the lesion. Notably, on gadolinium-enhanced sequences, the mass showed no uptake of contrast, consistent with an avascular structure. This absence of contrast enhancement strongly favored a thrombus over a vascular tumor, since tumors typically enhance due to their blood supply. No other abnormalities were seen in the cardiac chambers, valves, or mediastinal structures.

### Laboratory investigations

Except for a slightly elevated D-dimer, coagulation parameters were within normal limits. There was no evidence of a prothrombotic state. Additionally, no signs of infection or inflammatory processes were observed.

### Electrocardiogram (ECG)

Serial ECGs demonstrated a sinus rhythm appropriate for age, with no pauses, atrioventricular block, arrhythmias, or other electrocardiographic abnormalities.

The imaging findings with the lack of MRI contrast uptake were most consistent with an organized thrombus. Given the strong suspicion of an intracardiac thrombus and the need to prevent appositional growth, therapeutic anticoagulation with enoxaparin was initiated. However, after 16 days of treatment, serial ultrasounds revealed no change in the size or structure of the atrial mass. Due to the mass's persistence, signs of calcification, the uncertain nature of the lesion, and the continued risk of embolization, a multidisciplinary team decided to proceed with surgical removal. This decision also took into account the potential risks associated with prolonged anticoagulation if the mass were ultimately found to be non-thrombotic.

**Figure 3:**
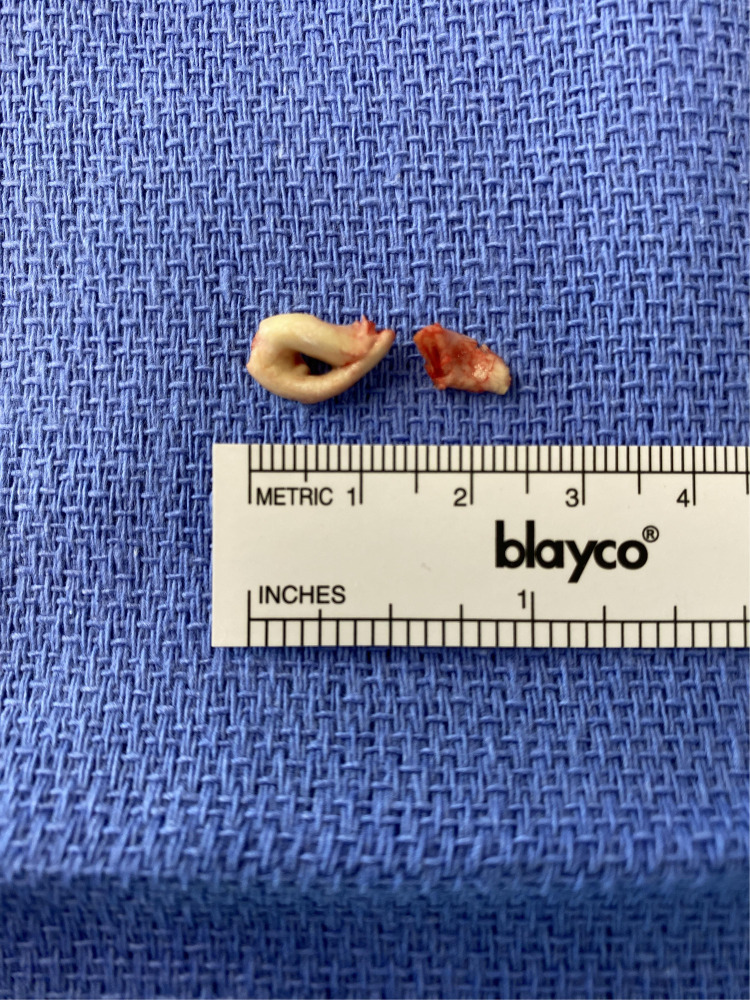
Gross specimen of right atrial mass after open surgical resection.

At approximately three months of age, the infant was transferred to a specialized pediatric cardiac center for surgical management. An open surgical resection of the right atrial mass was performed via median sternotomy and cardiopulmonary bypass. Intraoperatively, the mass was found attached to the interatrial septum and was completely excised (Figure 3). The surgery and anesthesia were uneventful. Postoperatively, anticoagulation was continued for three months with a transition to oral rivaroxaban for ongoing thromboprophylaxis.

### Histopathological findings

The excised atrial mass was sent for histopathological analysis. Gross examination showed an ovoid, firm, pale brown tissue piece with areas of surface calcification. Microscopic examination revealed fragments of endocardial tissue. Subendocardially, there were multiple areas of endocardium-adherent fibrinous material with prominent dystrophic calcification, confirmed by von Kossa staining. The lesions displayed abundant myofibroblasts and histiocytes, along with active neoangiogenesis, evidenced by numerous thin-walled capillaries. These features are consistent with organization and revascularization. No florid inflammatory infiltrate was observed, and there were no atypical or malignant cellular structures. The morphological findings are characterized by fibrinous material with marked dystrophic calcification and signs of organization and revascularization. These changes were consistent with a regressed mass lesion. A definitive distinction between a degeneratively altered thrombus and a previously existing (benign) neoplastic process with extensive degeneration was not possible based on the available material. This was confirmed by a second reference histopathology center. No histological evidence of malignancy was identified.

### Outcome

Following surgical removal of the mass, the infant recovered well. Echocardiography performed postoperatively and on follow-up confirmed the complete absence of any residual atrial mass and demonstrated unimpaired biventricular function. Over the next several weeks, the infant continued to thrive. The patient was discharged home on anticoagulation therapy and remained on oral anticoagulant for a total duration of three months post-surgery, after which it was electively discontinued under hematology guidance since no underlying thrombophilia was identified.

At the latest follow-up four months after surgery, the infant was feeding and growing well with no recurrence of the cardiac mass on echocardiography. Neurodevelopmentally, the child was age-appropriate. Given the indeterminate nature of the excised lesion, vigilant long-term follow-up was implemented with periodic echocardiographic surveillance to monitor for any new intracardiac masses or recurrence. To date, no further cardiac abnormalities have been detected. The case underscores the importance of ongoing monitoring in such scenarios, as the true identity of the mass (thrombus vs. tumor) remained unresolved. Fortunately, the outcome thus far has been favorable, and the child has not exhibited any complications related to the intra-atrial mass or its removal.

## Discussion

This case illustrates the challenges in diagnosing and managing neonatal cardiac masses, particularly the difficulty in differentiating an intracardiac thrombus from a primary tumor. In neonates, both thrombi and tumors are rare, and they can present with similar clinical and imaging features, creating a diagnostic challenge. Small, mobile masses in the atrium could represent either a bland thrombus or a tumor, like a myxoma (17). Our patient had no obvious risk factors to strongly suggest a thrombus, such as an intra-atrial indwelling catheter or sepsis, and the initial discovery was incidental, which raised the suspicion of a tumor. Indeed, there are reports of atrial thrombi in infants mimicking tumors in both presentation and echocardiographic appearance (18). On the other hand, some benign tumors (e.g., atrial myxomas) can be misidentified as thrombi.

Transthoracic echocardiography provided the first visualization of the mass and allowed serial monitoring of its size, mobility, and hemodynamic impact. The echocardiographic appearance (inhomogeneous texture with a stalk and no invasion of cardiac tissue) did not definitively distinguish between a clot and a tumor, but it provided clues and baseline information. Cardiac MRI added valuable tissue characterization: the lack of gadolinium enhancement and absence of myocardial infiltration were strongly suggestive of a thrombus, as thrombi are avascular and typically do not enhance with gadolinium (19). While our imaging studies favored a thrombus, they could not provide absolute certainty. A hematology consult was obtained, and empiric therapeutic anticoagulation was started, as resolving a thrombus non-surgically would avoid surgery on a very low-weight infant.

In neonatal patients, however, the threshold for surgical intervention in the case of an intracardiac mass is low when there is significant ambiguity or risk. In this case, the mass persisted despite adequate anticoagulation for 16 days. Given the potential for emboli or sudden blockage of circulation, the possible life-threatening sequelae (pulmonary embolus or paradoxical embolism) demand early surgical removal of the mass (20–22). In pediatric myxomas, for instance, >70% of patients suffer embolic events if not treated (23). In our case, the multidisciplinary team opted for surgery both to eliminate the immediate risk and to obtain tissue for definite diagnosis.

Despite complete excision and thorough analysis, pathology could not definitively distinguish between an organized thrombus and a regressed benign tumor. This highlights an important nuance: in certain scenarios, thrombi and tumors can closely resemble each other under the microscope. Organized thrombi can undergo fibrotic changes and even develop myxoid (mucinous) areas, potentially mimicking the myxomatous stroma of some cardiac tumors (24).

Conversely, benign tumors like cardiac myxomas may undergo degeneration, hemorrhage, or calcification, especially if they have been present for a while, making parts of them look like old thrombus material (25). In our case, the absence of any definitive tumor cells leans towards the lesion being an organized thrombus. However, we must consider that certain neonatal tumors (for instance, cardiac hemangiomas or teratomas) can sometimes involute or thrombose spontaneously (26).

Long-term follow-up is an essential component in cases of neonatal cardiac masses, particularly when the exact pathology remains unclear. We plan to continue periodic cardiac evaluations for our patient. Literature on outcomes of neonatal cardiac surgeries supports regular follow-up, as even in cases with a definite diagnosis (tumor or thrombus), infants benefit from monitoring for cardiac function, potential recurrence, and developmental progress (16).

## Conclusion

Early detection and thorough evaluation of an intracardiac mass in a neonate are crucial for guiding management and improving outcomes. This case demonstrates that the integration of echocardiography and advanced imaging modalities like cardiac MRI is valuable in assessing such masses, as each modality contributes complementary information toward the differential diagnosis. However, even with state-of-the-art imaging, the true nature of a cardiac mass may remain uncertain until surgical excision and histopathological examination are performed – and, in rare instances, even the pathology may be inconclusive. In our patient, the intra-atrial mass was initially managed as a thrombus, yet the diagnosis could not be definitively secured as a thrombus or tumor, highlighting the inherent diagnostic limitations. In general, if a cardiac mass persists despite anticoagulation therapy, or if there is significant diagnostic uncertainty or notable clinical risks—such as bleeding, embolism, arrhythmias, or sudden cardiac death—prompt surgical intervention is warranted. Early removal of the mass can help prevent serious complications, including embolization or intracardiac obstruction. The infant in our report had an excellent outcome, with long-term follow-up currently ongoing.

## Data Availability

The raw data supporting the conclusions of this article will be made available by the authors, without undue reservation.

## References

[1] Nadas AS Ellison RC. Cardiac tumors in infancy. Am J Cardiol. (1968) 21(3):363–6. 10.1016/0002-9149(68)90140-9 4866645

[2] Sillesen A Joergensen FS Petersen OB Zingenberg HJ Axelsson Raja A Voegg ROB The prevalence of congenital cardiac tumours in unselected newborns. Eur Heart J. (2023) 44(Supplement_2):2165. 10.1093/eurheartj/ehad655.1895

[3] Webb DW Thomas RD Osborne JP. Cardiac rhabdomyomas and their association with tuberous sclerosis. Arch Dis Child. (1993) 68(3):367–70. 10.1136/adc.68.3.367 8466239 PMC1793857

[4] Delmo Walter EM Javier MF Sander F Hartmann B Ekkernkamp A Hetzer R. Primary cardiac tumors in infants and children: surgical strategy and long-term outcome. Ann Thorac Surg. (2016) 102(6):2062–9. 10.1016/j.athoracsur.2016.04.057 27344282

[5] Arciniegas E Hakimi M Farooki ZQ Truccone NJ Green EW. Primary cardiac tumors in children. J Thorac Cardiovasc Surg. (1980) 79(4):582–91. 10.1016/S0022-5223(19)37924-3 7359937

[6] Isaacs H. Fetal and neonatal cardiac tumors. Pediatr Cardiol. (2004) 25(3):252–73. 10.1007/s00246-003-0590-4 15360117

[7] Dianzumba SB Char G. Large calcified right atrial myxoma in a newborn. Rare cause of neonatal death. Br Heart J. (1982) 48(2):177–9. 10.1136/hrt.48.2.177 7093087 PMC481223

[8] Dominguez C Perkins A Duque A Bravo V. Primary cardiac tumors in infancy: a case report and literature review. Acad Forensic Pathol. (2017) 7(1):112–8. 10.23907/2017.013 31239963 PMC6474475

[9] Gover A Sharif D Yaniv L Riskin A. Intracardiac thrombi in preterm infants-A case study and review of the literature. Diagnostics (Basel). (2023) 13(4):764. 10.3390/diagnostics13040764 36832252 PMC9955841

[10] Khizroeva J Makatsariya A Vorobev A Bitsadze V Elalamy I Lazarchuk A The hemostatic system in newborns and the risk of neonatal thrombosis. Int J Mol Sci. (2023) 24(18):13864. 10.3390/ijms241813864 37762167 PMC10530883

[11] Angelo Claudio M Saracco P. The thrombotic risk of the newborn. In: Buonocore G Bracci R Weindling M, editors. Neonatology. Cham: Springer International Publishing (2018). p. 1–15.

[12] Agarwal S Abdelghani E Stanek JR Sankar A Cua CL Kerlin BA Intracardiac thrombi in pediatrics: anticoagulation approach and treatment outcomes. Res Pract Thromb Haemost. (2023) 7(8):102266. 10.1016/j.rpth.2023.102266 38193068 PMC10772888

[13] Goitein O Slonimasky E Hamdan A Salem Y Di Segni E Konen O The accuracy of cardiac MRI in differentiating between intra cardiac tumors and thrombi. J Cardiovasc Magn Reson. (2015) 17:Q70. 10.1186/1532-429X-17-S1-Q70

[14] Parato VM Nocco S Alunni G Becherini F Conti S Cucchini U Imaging of cardiac masses. J Cardiovasc Echogr. (2022) 32(2):65–75. 10.4103/jcecho.jcecho_18_22 36249434 PMC9558634

[15] Medina Perez M Lichtenberger JP Huppmann AR Gomez M Ramirez Suarez KI Foran A Cardiac and pericardial neoplasms in children: radiologic-pathologic correlation. Radiographics. (2023) 43(9):e230010. 10.1148/rg.230010 37561644

[16] Morin CE Griffin LM Beroukhim RS Caro-Domínguez P Chan S Johnson JN Imaging of pediatric cardiac tumors: a COG diagnostic imaging committee/SPR oncology committee white paper. Pediatr Blood Cancer. (2023) 70 Suppl 4(Suppl 4):e29955. 10.1002/pbc.29955 36083866 PMC10641876

[17] Sheen A de Oliveira ER Kim RW Parham D Lakshmanan A. Atrial thrombus in a neonate: a diagnostic challenge. AJP Rep. (2015) 5(1):e18–21. 10.1055/s-0034-1396567 26199791 PMC4502629

[18] Hu G Song F. Atrial thrombus or atrial myxoma? Preliminary analysis of echocardiographic findings of a case series. Curr Cardiol Rev. (2024) 20(5):e250424229325. 10.2174/011573403X281926240417110330 38676477 PMC11337610

[19] O'Donnell DH Abbara S Chaithiraphan V Yared K Killeen RP Cury RC Cardiac tumors: optimal cardiac MR sequences and spectrum of imaging appearances. AJR Am J Roentgenol. (2009) 193(2):377–87. 10.2214/AJR.08.1895 19620434

[20] Pawar RD Garg A Dolas A. Cardiac distress in an infant—giant right atrial myxoma obstructing right ventricular inflow and causing cardiac arrest. Med J Dr. D.Y. Patil Vidyapeeth. (2023) 16(Suppl 1):S154–6. 10.4103/mjdrdypu.mjdrdypu_595_22

[21] Samanidis G Kanakis M Bobos D Kousi T Dimitropoulou M Karafotia A Right atrial thrombus mimicking cardiac tumor in a neonate. Clin Case Rep. (2020) 8(12):3642–4. 10.1002/ccr3.3396 33364017 PMC7752545

[22] Iezzi F Quarti A Capestro A Pozzi M. Obstructive neonatal atrial myxoma. Int J Surg Case Rep. (2017) 37:57–9. 10.1016/j.ijscr.2017.05.036 28641192 PMC5479953

[23] Rafi Khourgami M Ershad A Mozaffari K. Left atrial myxoma misdiagnosis as infective endocarditis: a case report. Arch Pediatr Infect Dis. (2020) 9(3):e108029. 10.5812/pedinfect.108029

[24] AbdullGaffar B Waslewski K. Myxoid emboli. Int J Surg Pathol. (2018) 26(7):609–16. 10.1177/1066896918768029 29623727

[25] Bussani R Castrichini M Restivo L Fabris E Porcari A Ferro F Cardiac tumors: diagnosis, prognosis, and treatment. Curr Cardiol Rep. (2020) 22(12):169. 10.1007/s11886-020-01420-z 33040219 PMC7547967

[26] Uzun O Wilson DG Vujanic GM Parsons JM de Giovanni JV. Cardiac tumours in children. Orphanet J Rare Dis. (2007) 2:11. 10.1186/1750-1172-2-11 17331235 PMC3225855

